# Genetic Variants in microRNA Target Sites of 37 Selected Cancer-Related Genes and the Risk of Cervical Cancer

**DOI:** 10.1371/journal.pone.0086061

**Published:** 2014-01-22

**Authors:** Yang Mi, Lijuan Wang, Lu Zong, Meili Pei, Qingyang Lu, Pu Huang

**Affiliations:** 1 Obstetrical department, Maternal and Child Health Hospital of Shaanxi Province, Xi'an, ShaanXi, Peoples' Republic of China; 2 Department of Obstetrics and Gynecology, The First Affiliated Hospital of Xi'an Jiaotong University College of Medicine, Xi'an, ShaanXi, Peoples' Republic of China; 3 Department of Pathology, Liaocheng People's Hospital, Liaocheng, ShanDong, Peoples' Republic of China; Duke Cancer Institute, United States of America

## Abstract

**Objectives:**

Single nucleotide polymorphisms (SNPs) in putative microRNA binding sites (miRSNPs) modulate cancer susceptibility via affecting miRNA binding. Here, we sought to investigate the association between miRSNPs and cervical cancer risk.

**Methods:**

We first genotyped 41 miRSNPs of 37 cancer-related genes in 338 patients and 334 controls (Study 1), and replicated the significant associations in 502 patients and 600 controls (Study 2). We tested the effects of miRSNPs on microRNA-mRNA interaction by luciferase reporter assay.

**Results:**

Five SNPs displayed notable association with cervical cancer risk in Study 1. Only *IL-16* rs1131445 maintained a significant association with cervical cancer (CT/CC vs. TT, adjusted OR = 1.51, *P* = 0.001) in Study 2. This association was more evident in the combined data of two studies (adjusted OR = 1.49, *P* = 0.00007). We also found that miR-135b mimics interacted with *IL-16* 3′-UTR to reduce gene expression and that the rs1131445 T to C substitution within the putative binding site impaired the interaction of miR-135b with *IL-16* 3′-UTR. An ELISA indicated that the serum IL-16 of patients with cervical cancer was elevated (vs. controls, *P* = 0.001) and correlated with the rs1131445 genotype. Patients who carried the rs1131445 C allele had higher serum IL-16 than non-carriers (*P*<0.001).

**Conclusions:**

These results support our hypothesis that miRSNPs constitute a susceptibility factor for cervical cancers. rs1131445 affects *IL-16* expression by interfering with the suppressive function of miR135b and this variant is significantly associated with cervical cancer risk.

## Introduction

Cervical cancer, the third most common cancer in women [1], is mainly caused by human papillomavirus (HPV) infection [2]. Although HPV infections are prevalent in sexually active women, only a minority of infections persist and develop into cervical intraepithelial neoplasia grade 2/3 (CIN 2/3) or even cervical cancer [3]. Several cofactors affect the transition from initial HPV infection to cervical cancer, including life-style, host immune response, and genetic susceptibility [4], [5]. The important roles of genetic factors in cervical cancer pathogenesis are prompted by the findings that cervical cancer exhibits a significant familial clustering and the first degree relatives of patients have double risk of developing cancer [6]. A few risk-modulating variants for cervical cancer have been identified by a candidate gene association study. The genes that are associated with cervical cancer susceptibility are involved in DNA repair, cellular cycle and apoptosis, cell proliferation and differentiation, the human leukocyte antigen system and immune responses [7], [8].

Recent compelling evidence indicates that polymorphisms in microRNA (miRNA) binding sites of cancer-related genes are an important source that harbors the causative genetic variants of cancer [9]. miRNA, often 19–25 nucleotide-length noncoding RNA, binds within the 3′ untranslated region (UTR) of transcriptions of targeted genes to inhibit and even abolish its translation to protein. It is estimated that approximately 30% of human genes are regulated by miRNAs [10]. Gene deregulation is one of the key mechanisms by which cells can progress to cancer [11]. miRNA can participate in the carcinogenesis via post-transcriptional regulation of many cancer-related genes, including oncogenes and tumor suppressor genes. The aberrant expression of miRNAs contributes to the etiology of multiple cancers, including cervical cancer [12]. miRNA profile analysis of cervical cancer revealed that miRs-9, 21, 135b, 146a, 199a, 203 and 205 are frequently overexpressed in cancer tissues or cell lines [12]. Moreover, miR-205 acts as an oncogene to promote cell proliferation and migration of human cervical cancer cells [13]. In contrast, miR-214 directly targets the GALNT7 gene to suppress growth and invasiveness of cervical cancer cells by functioning as a tumor suppressor a tumor suppressor [14]. The base pair change within the miRNA-target region of cancer-related genes could destroy or create a binding site or alter the binding affinity and post-transcriptional control of mRNA by miRNA and, therefore, could affect the transformation from normal cells to cancer cells [9].

Bioinformatics analysis has identified many SNPs that are located in the putative miRNA binding sites of cancer candidate genes and that significantly affect the binding energy of putative miRNA::mRNA duplexes [15]. A small fraction of them have been further proved to alter the expression of target genes by the luciferase reporter assay and display a significant association with susceptibility to cancer [16], [17]. Landi et al. screened out 57 SNPs within miRNA-binding sites from 104 candidate genes for colorectal cancer, found eight SNPs of them showing various Gibbs free energy of binding between the two alleles of each SNP, and finally demonstrated a SNP, rs17281995, within the miRNA-binding sites of CD86, as the risk-modulating variant for colorectal cancer [16]. Using a similar research strategy, SNPs within miRNA binding sites of other important cancer candidate genes, such as DNA repair genes [17], integrin genes [18] and caspase genes [19], have also been identified and further evaluated as susceptibility variants for bladder cancer, breast cancer, colorectal cancer, and head and neck cancer risk. Recently, Shi et al. demonstrated that the rs11064 G allele within the *TNFAIP8* gene weakens the binding affinity of miR-22 to the TNFAIP8 3′- UTR and is associated with an increased risk of cervical cancer [20], which suggests that miRNA-binding sites are also the hot spots that harbor cervical cancer susceptibility variants.

In the present study, we sought to identify new risk variants for cervical cancer among miRNA-target sites in cancer-related genes. By reviewing the literature and bioinformatics databases, we found 37 cancer-related genes with variations in putative 3′-UTR miRNA binding sites. The association strength of these variants with cervical cancer was assessed by a two-stage case-control study. For the associated variant, its functional influence on miRNA-mediated expression regulation of the target genes was investigated using the luciferase reporter assay.

## Materials and Methods

### 1 Study population

We recruited 840 patients with cervical cancer from the patients who received inpatient treatment in three hospitals between May 2008 and April 2012. Among them, 338 subjects from Liaocheng People's Hospital (LCPH) were used in first stage study (Study 1), which investigated all of the selected SNPs, and 502 subjects from The First Affiliated Hospital of Xi'an Jiaotong University (TFAHXJTU) and Maternal and Child Health Hospital of Shaanxi Province (MCHHSP) were used in second stage study (Study 2), which was to replicate the noteworthy findings (*P*<0.05) from Study 1. All of the patients were diagnosed by histological examination of biopsy under colposcope and/or resected tissues. The clinical stage and histological type of cervical cancer of patients were collected from medical records and are shown in Table 1. A total of 934 healthy controls were recruited from the women who routinely attended physical examination in these three hospitals, 334 subjects from LCPH in Study 1 and 600 subjects from TFAHXJTU and MCHHSP in Study 2. All of the controls had no history of neoplastic or cancer-associated illness, and they had normal cervical cytology in at least two consecutive annual examinations. All of the subjects were genetically unrelated, and their age, race, smoking status, socio-economic status, and marital status were also investigated by reading medical records or by interviews. This study was approved by the Medical Ethics Committees of LCPH, TFAHXJTU, and MCHHSP and all of the participants gave informed written consent.

**Table 1:** The characteristics of patients with cervical cancer and cancer-free controls.

	Study 1			Study 2			All	subjects	
Characteristics	Controls (n = 334)	Cases (n = 338)	*P* b	Controls (n = 600)	Cases (n = 502)	*P*	Controls (n = 934)	Cases (n = 840)	*P*
**Age** a **, year (Mean**±**SD)**	47.2±10.8	46.5±8.7	0.355	48.4±11.2	47.5±9.5	0.262	48.0±11.1	47.1±9.2	0.065
**Race, n (%)**									
Han Chinese	290 (86.8)	285 (84.3)	0.355	538 (89.7)	453 (90.2)	0.753	828 (88.7)	738 (87.9)	0.604
Hui Chinese	44 (13.2)	53 (15.7)		62 (10.3)	49 (9.8)		106 (11.3)	102 (12.1)	
**Smoking**									
Never	305 (91.3)	313 (92.6)	0.540	562 (93.7)	448 (89.2)	0.008	867 (92.8)	761 (90.6)	0.088
Ever	29 (8.7)	25 (7.4)		38 (6.3)	54 (10.8)		67 (7.2)	79 (9.4)	
**Education**									
JHS and below	189 (56.6)	201 (59.5)	0.550	314 (52.3)	282 (56.2)	0.717	503 (53.9)	483 (57.5)	0.488
SHS and above	145 (43.4)	137 (40.5)		286 (47.7)	220 (43.8)		431 (46.1)	357 (42.5)	
**Age at primiparity**									
≤24 years	206 (63.6)	219 (66.6)	0.424	390 (67.0)	365 (73.1)	0.031	596 (65.8)	575 (70.5)	0.038
>24 years	118 (36.4)	110 (33.4)		192 (33.0)	131 (26.9)		310 (34.2)	241 (29.5)	
Missing or nulliparous	10	9		18	15		28	24	
**Marital status**									
Never married	6 (1.9)	4 (1.2)	0.195	15 (2.7)	11 (2.3)	0.145	21(2.4)	15 (1.8)	0.035
Married only once	259 (83.5)	264 (79.3)		453 (80.3)	370 (76.0)		712 (81.5)	634 (77.4)	
≥2 times	45 (14.5)	65 (19.5)		96 (17.0)	106 (21.7)		141 (16.1)	171 (20.9)	
Missing	24	5		36	15		60	20	
**HPV infection status**									
HPV +	106 (31.7)	296 (87.6)	**<0.001**	229 (38.2)	420 (83.7)	**<0.001**	335 (35.9)	716 (85.2)	**<0.001**
HPV -	228 (68.3)	42 (12.4)		371 (61.8)	82 (16.3)		599 (64.1)	124 (14.8)	
**Clinical stage** c									
I		247 (73.1)			365 (72.7)			612 (72.9)	
II		69 (20.4)			118 (23.5)			187 (22.3)	
II I/IV		22 (6.5)			19 (3.8)			41 (4.9)	
**Histology**									
SC		280 (82.8)			398 (79.3)			678 (80.7)	
Adenocarcinoma		44 (13)			75 (14.9)			119 (14.2)	
Other		14 (4.1)			29 (5.8)			43 (5.1)	

a Age of diagnosis for cases and age when they participated in this study for controls.

b Statistically significant results with an FPRP value <0.2 are marked in bold.

c Based on International Federation of Gynecology and Obstetrics classification system.

Abbreviation: SD, standard deviation; JHS, junior high school; SHS, senior high school; SC: Squamous carcinoma.

### 2 Polymorphism selection and genotyping

Previous studies have systematically screened the SNPs in miRNA binding sites of many cancer-related genes. Briefly, they first screened cancer-related genes by database and literature searches, and then, they identified the putative miRNA target sites in these genes by specialized algorithms (such as miRBase, miRanda, PicTar and TargetScan) and selected the SNPs that reside on the miRNA-binding sites by dbSNP search and BLAST, finally identifying the SNPs whose alleles make the target sequence have significantly differential binding energy to miRNA in a computer predictive model [16], [18]. Because many cancer-related genes are shared by multiple cancers [21], we selected the SNPs from the previous literature that covers the topics about miRNA-binding site/miRNA target sites, polymorphisms and cancer/tumor. The MedLine/PubMed database was searched to find literature that was published from 2000 January to 2013 January. We lastly selected 41 SNPs in predicted miRNA-binding sites within 37 cancer-related genes with minor allele frequencies greater than 0.1 in the Chinese Han population (Table 2). Genomic DNA was extracted from peripheral blood leukocytes using the Blood DNA Midi Kit (Omega Biotech, Norcross, USA), according to the manufacturer's protocol. The selected SNPs were genotyped in patients and controls using MALDI-TOF within the MassARRAY system (Sequenom Inc., San Diego, CA, USA) [22].

**Table 2:** The candidate cancer-related genes, the selected SNPs, the miRNAs whose binding to target mRNAs could be altered by the corresponding base substitution, and the frequency distributions of selected SNPs in the cases and controls in Study 1.

Gene	Function involved	dbSNP ID	Chromosome/Position	The binding miRNA^a^	Homozygous major allele (controls/cases)	Heterozygote (controls/cases)	Homozygous major allele(controls/cases)	Chi-squarevalue	*P* value^b^
REV3L	DNA metabolism and repair	rs465646 A/G	6/111620758	**miR-25**	225/253	98/80	10/3	7.216	**0.027**
RYR3	Regulation of cytosolic calcium	rs1044129 A/G	15/34158266	**miR-367**	116/110	173/188	44/39	1.06	0.589
BIRC5	Regulation of apoptosis	rs2239680 T/C	17/76219783	**miR-335**	206/183	116/127	10/25	8.27	**0.016**
NBS1	DNA repair	rs2735383 G/C	8/90947269	**miR-629**	109/115	150/162	74/60	2.061	0.357
RAP1A	Oncogene	rs6573 C/A	1/112255389	**miR-196a**	196/205	118/113	18/20	0.362	0.835
FGF2	Mitosis, angiogenesis and tumor growth	rs1476215 T/A	4/123818271	miR-196a	277/259	50/56	5/23	12.463	**0.002**
TGFA	Cell proliferation and differentiation	rs473698 G/C	2/70411736	miR-495	165/165	130/141	39/32	1.113	0.573
DGCR8	microRNAs biogenesis	rs3757 G/A	22/20099331	miR-523	222/230	100/99	10/9	0.146	0.93
ITGA6	Cell proliferation, migration and survival	rs17664 G/A	2/173369231	miR-152	195/213	112/108	27/16	3.667	0.16
ITGB3	Cell proliferation, migration and survival	rs3809865 A/T	17/45388586	miR-26b	201/213	110/115	23/10	5.557	0.062
ITGB5	Cell proliferation, migration and survival	rs2675 A/C	3/124482113	miR-504	233/246	94/83	5/7	1.346	0.51
LIG3	DNA Repair	rs4796030A/C	17/33330150	miR-612	123/127	156/172	55/39	3.544	0.17
BRCA1	DNA Repair	rs12516 C/T	17/41196408	miR-874	159/153	133/153	42/32	2.842	0.242
BRCA1	DNA Repair	rs8176318 C/T	17/41197274	miR-328	138/162	151/142	45/34	3.704	0.157
XPC	DNA repair	rs2229090 C/G	3/14187345	miR-1225-3p	167/173	135/134	32/31	0.102	0.95
RPA1	DNA repair	rs9914073 A/G	17/1801592	miR-548c-3p	135/147	157/158	42/33	1.57	0.456
RPA2	DNA repair	rs7356 A/G	1/28218100	miR-3149	87/96	178/170	68/72	0.704	0.703
GTF2H1	DNA repair	rs4596 G/C	11/18388128	miR-518a-5p	101/88	180/191	53/59	1.518	0.468
ERCC4	DNA Repair	rs4781563 G/A	16/14045399	miR-2355-3p	195/206	122/108	17/21	1.573	0.455
CASP3	Apoptosis	rs1049216 C/T	4/185550089	miR-181	187/193	131/131	16/14	0.204	0.903
CASP6	Apoptosis	rs1042891 C/T	4/110610086	miR-944	96/105	155/151	83/82	0.438	0.804
CASP7	Apoptosis	rs1127687 G/A	10/115490109	miR-136	191/189	120/125	23/24	0.11	0.946
CASP7	Apoptosis	rs10787498 T/G	10/115489650	miR-19a	163/179	149/144	20/15	1.495	0.474
CASP7	Apoptosis	rs12247479 G/A	10/115490060	miR-345	258/271	69/61	7/6	0.865	0.649
CASP7	Apoptosis	rs4353229 T/C	10/115489589	miR-224	113/111	175/183	46/44	0.217	0.897
E2F1	Cell proliferation and apoptosis	rs3213180 G/C	20/32263624	miR-1182	178/154	128/135	26/49	8.922	**0.012**
IL16	Immune response/inflammation	rs1131445 T/C	15/81601782	miR-135a	126/100	169/180	39/58	7.036	**0.030**
PTGER4	Immune response/inflammation	rs16870224 G/A	5/40692940	miR-9,	243/234	83/86	7/17	4.366	0.113
NOD2	Immune response/inflammation	rs3135500 G/A	16/50766886	miR-158	164/178	129/132	41/28	3.033	0.219
PLA2G2A	Phospholipid metabolism	rs11677C/T	1/20301964	miR-187	213/196	109/121	12/20	3.319	0.19
ALOX15	Linoleic acid metabolism	rs916055 T/T	17/4138208	miR-588	130/122	155/151	49/65	2.528	0.283
IL-23 R	Immune response/inflammation	rs10889677 A/C	1/67725120	**Let-7e**	189/180	131/141	14/17	0.854	0.653
TNFAIP2	Apoptosis	rs8126 T/C	14/103603569	**miR-184**	144/147	138/158	52/33	5.606	0.061
MTMR3	Apoptosis	rs12537 C/T	22/30423460	**miR-181a**	224/233	94/93	16/12	0.73	0.694
HOXB5	Cellular morphogenesis and differentiation	rs9299 A/G	17/46669430	**miR-7**	92/101	174/188	68/49	4.023	0.134
TYMS	DNA replication and repair	rs699517 T/C	18/673016	miR-498	163/155	130/153	41/30	3.751	0.153
EFNA1	Tumor development and maintenance	rs12904 A/G	1/155106697	**miR-200c-3p**	210/218	115/106	9/14	1.579	0.454
MYCL1	A highly plausible oncogene	rs3134615 C/T	1/40362066	**miR-1827**	271/283	57/52	6/3	1.466	0.481
TGFBR1	Cell proliferation, migration and apoptosis	rs334348 A/G	9/101912471	**miR-628-5p**	104/91	152/143	78/104	4.832	0.089
TP53BP2	Apoptosis	rs17739 C/T	1/223967953	miR-129-5p	182/161	127/147	25/30	3.176	0.204
TP53INP1	A tumor suppressor	rs7760 T/G	8/95938422	miR-330-5p	241/254	81/74	12/10	0.816	0.665

a The binding miRNAs that are supported by functional experiment, such as the luciferase reporter assay, are in boldface.

b *P* value was calculated using a χ^2^ test with 2 d.f. P≤0.05 are in boldface.

### 3 HPV genotyping

Human HPV from cervical scraping smear and/or resected tissues was genotyped using the HPV GenoArray test kit (HybriBio Ltd, Guangdong, China). First, HPV DNA was amplified with the L1 consensus HPV primers MY09/MY11 and HMB01. Then, the PCR product processed flow-through hybridization in the manner of a macroarray format with a nylon membrane onto which HPV genotype-specific oligonucleotide probes were immobilized. This technique can identify 21 HPV genotypes, including 5 low-risk types (6, 11, 42, 43, and 44), 14 high-risk types (16, 18, 31, 33, 35, 39, 45, 51, 52, 56, 58, 59, 66, and 68), and 2 intermediate-risk types (CP8304 and 53).

### 4 Serum IL-16 levels

The patients' and controls' blood samples were collected between 8 and 10 a.m. The sera were centrifuged at 3000 rpm for 10 min and frozen at −80°C. Serum IL-16 levels were measured by commercially available enzyme-linked immunosorbent assay (ELISA) kits (PIERCE Endogen) according to the manufacturer's instructions. The minimum level of detection for IL-16 was 0.10 pg/ml. No cross-detection of other cytokines was observed. The intra-assay variation was <10%.

### 5 Cell culture

Human cervical cancer cell lines, HeLa [23] and SiHa [24], were purchased from the Type Culture Collection of the Chinese Academy of Sciences (Shanghai, China). These cells were cultured in Dulbecco modified Eagle medium supplemented with 100 U/ml penicillin, 100 mg/ml streptomycin, and 10% fetal bovine serum and were grown at 37°C in a humidified 5% carbon dioxide incubator.

### 6 Transient transfections and luciferase assays

The 3′-UTR of IL-6 mRNA, including the C allele or T allele of rs1131445, were amplified using the primers of 5′-TGGCCTGGGCCTCCTCACAA-3′ (forward) and 5′- CTCCACCACCCTTCCCTA -3′ (reverse). The amplified fragments were then cloned into the downstream of the firefly luciferase gene in the pGL3-basic vector (Promega, Madison, USA). These recombination plasmids were sequenced to confirm their accuracy. For the luciferase reporter assays, HeLa and SiHa was plated in 24-well dishes to be co-transfected with 100 ng of pGL3-C or pGL3-T constructs, 50 pmol/µL of chemically synthesized miRNAs miR-135b (GenePharma, Shanghai, China) or negative control miRNA using Lipofectamine 2000 transfection reagent (Invitrogen, Carlsbad, USA). In each transfection, 20 ng of pRL-TK plasmid (Promega, Madison, USA) was used to correct for transfection efficiency. Each transfection was conducted in triplicate. Cells were collected 48 hours after transfection, and luciferase activity was measured with a dual-Luciferase reporter assay system (Promega, Madison, USA) and normalized against the activity of the Renilla luciferase gene.

### 7. Statistical analysis

We calculated the statistical power as previously described [25]. A χ^2^ test was performed to make a comparison of the categorical variables, including the genotype frequency and some demographic characteristics. Student's t-test, Mann–Whitney U-test or analysis of variance were conducted to compare the continuous variables, such as the age, serum IL-16 level and reporter expression level, when appropriate. The associations between the *IL-16* variants and the cervical cancer risk were estimated by computing the ORs and their 95% CIs from multivariate logistic regression analyses with an adjustment for the age, body mass index, smoking status and family history of cancer. To avoid a spurious association caused by multiple tests, the false positive report probability (FPRP) was calculated according to the method described by Wacholder et al. [26]. We set 0.2 as an FPRP threshold and assigned a prior probability of 0.01 to detect an OR of 1.50 (for the risk effects) or 0.67 (for the protective effects) for an association with the SNPs or clinico-demographic features. The Hardy–Weinberg equilibrium (HWE) of the SNPs among the controls was evaluated by the χ^2^ test. All of the statistical analyses were performed by SPSS (version 13.0, Chicago, IL, USA). A value of *P*<0.05 was considered to be significant (two-tailed).

## Results

### 1 Population characteristics

We analyzed altogether 840 patients with cervical cancer and 934 controls in Studies 1 and 2. The demographic and clinical characteristics of patients and controls are presented in Table 1. There was no significant difference in the distribution of the age, race, smoking status, educational level, and marital status between the controls and the cases in Study 1, Study 2 and all of the subjects combined. However, the cases were more likely to be younger at age at first delivery (*P* = 0.038) and with HPV infection (*P*<0.001) than the controls. HPV infection still remained significantly associated with cervical cancer after considering the FPRP that was less than 0.2. These two variables were further adjusted for any residual confounding effect in later multivariate logistic regression analyses.

### 2 Association of SNPs within miRNA-binding sites of cancer-related genes with cervical cancer risk

More than 99% of the samples were genotyped successfully for each SNP, and the replicate experiment for the randomly selected 300 samples acquired completely consistent genotype data with the original analysis. Table 2 shows the list of selected SNPs within the miRNA-binding sites in the 3′-UTR of 37 cancer-related genes, the binding miRNA that they potentially affect, and their genotype frequencies and associations with cervical cancer in Study 1. All of the observed genotype distributions among the controls of Study 1 were in line with the HWE. Among the 41 SNPs that were included in Study 1, five SNPs (rs465646 in *REV3L* gene, rs2239680 in *BIRC5* gene, rs1476215 in *FGF2* gene, rs3213180 in *E2F1* gene, rs1131445 in *IL-16* gene) exhibited a markedly different genotype distribution between the cases with cervical cancer and the controls (*P* = 0.003∼0.030). To determine whether true significant associations existed or whether there were spurious results, we performed a replicate to analyze these SNPs in another population that resides in Shaan Xi province of Northern China. In Study 2, their observed genotype frequencies were also consistent with HWE (Table 3). Moreover, we found that only *IL-16* rs1131445 continued to show a significant association with cervical cancer. The subjects with its TC or CC allele had an increased risk for cervical cancer (adjusted OR = 1.51, 95% CI 1.18–1.93, *P* = 0.001) with an adjustment for age, smoking status, educational level, age at primiparity, marital status, and HPV infection status. The association between rs1131445 and cervical cancer was more significant in data that combined Study 1 and 2 (adjusted OR = 1.49, 95% CI 1.22–1.81, *P* = 0.00007) with adjustment for the aforementioned variables. We then calculated the FPRP values for all of the observed significant associations. When the assumption of prior probability was 0.01, the association with the *IL-16* rs1131445 (TC/CC vs.TT) was still noteworthy in both Study 1 subjects (FPRP = 0.171) and all subjects (FPRP = 0.011). Given an OR of 1.5 at a nominal *P* = 0.05 for genotype frequencies ranging from 0.20 to 0.40, the statistical power of our study to detect an association of each SNP with cervical cancer in Study 2 and combined Study 1and 2 was estimated at 81.0–91.5% and 95.0–98.9%, respectively. The stratification analysis did not reveal a significant interaction of the demographic and clinical characteristics and the rs1131445 genotype on the cervical cancer risk (data not shown). Additionally, there were not significant correlations between the genotypes of rs1131445 and the clinical stage and histological type of cervical cancer (data not shown).

**Table 3:** Association of the selected SNPs from the Study 1 and an additional SNP in *IL-16* with the risk for cervical cancer in the Study 2.

				Study 2						Combined Study 1and 2		
Variant	Genotype	Controls	Cases		Logistic regression			Controls	Cases		Logistic regression	
		(n = 600) No. (%)	(n = 502) No. (%)	*P* value (2df)^a^	Adjusted OR (95%CI)	*P* value^b^		(n = 934) No. (%)	(n = 840) No. (%)	*P* value (2df)^a^	Adjusted OR (95%CI)	*P* value^b^
REV3L	AA	432 (72.0)	353 (70.6)	0.741	1.00 (referent)			657 (70.4)	606 (72.5)	0.599	1.00 (referent)	
rs465646	AG	153 (25.5)	131 (26.2)		1.05 (0.8∼1.38)	0.737		251 (26.9)	211 (25.2)		0.91 (0.74∼1.12)	0.392
	GG	15 (2.5)	16 (3.2)		1.31 (0.64∼2.69)	0.466		25 (2.7)	19 (2.3)		0.82 (0.45∼1.51)	0.532
	AG+GG	168 (28.0)	147 (29.4)		1.07 (0.82∼1.39)	0.609		276 (29.6)	230 (27.5)		0.91 (0.73∼1.11)	0.334
BIRC5	TT	353 (58.8)	277 (55.3)	0.199	1.00 (referent)			559 (60)	460 (55)	0.008	1.00 (referent)	
rs2239680	TC	215 (35.8)	185 (36.9)		1.10 (0.86∼1.41)	0.473		331 (35.5)	312 (37.3)		1.14 (0.94∼1.40)	0.179
	CC	32 (5.3)	39 (7.8)		1.56 (0.95∼2.55)	0.079		42 (4.5)	64 (7.7)		1.85 (1.23∼2.79)	0.004
	TC+CC	247 (41.2)	224 (44.7)		1.16 (0.91∼1.47)	0.236		373 (40)	376 (45)		1.22 (1.01∼1.47)	0.035
FGF2	TT	452 (75.3)	401 (79.9)	0.166	1.00 (referent)			729 (78.2)	660 (78.6)	0.018	1.00 (referent)	
rs1476215	TA	134 (22.3)	89 (17.7)		0.75 (0.55∼1.01)	0.059		184 (19.7)	145 (17.3)		0.98 (0.78∼1.23)	0.26
	AA	14 (2.3)	12 (2.4)		0.97 (0.44∼2.12)	0.933		19 (2)	35 (4.2)		0.87 (0.68∼1.11)	0.012
	TA+AA	148 (24.7)	101 (20.1)		0.77 (0.57∼1.03)	0.071		203 (21.8)	180 (21.4)		2.03 (1.15∼3.58)	0.858
E2F1	GG	325 (54.2)	277 (55.2)	0.869	1.00 (referent)			503 (54)	431 (51.3)	0.104	1.00 (referent)	
rs3213180	GC	224 (37.3)	180 (35.9)		0.95 (0.73∼1.22)	0.650		352 (37.8)	315 (37.5)		1.04 (0.85∼1.27)	0.669
	CC	51 (8.5)	45 (9.0)		1.03 (0.68∼1.59)	0.872		77 (8.3)	94 (11.2)		1.42 (1.02∼1.97)	0.032
	GC+CC	275 (45.8)	225 (44.8)		0.96(0.76∼1.21)	0.735		429 (46)	409 (48.7)		1.11 (0.92∼1.34)	0.264
IL16	TT	243 (40.5)	156 (31.1)	0.002	1.00 (referent)			369 (39.5)	256 (30.5)	0.00004	1.00 (referent)	
rs1131445	TC	284 (47.3)	258 (51.4)		1.42 (1.09∼1.85)	0.008		453 (48.5)	438 (52.1)		**1.40 (1.14**∼**1.72)**	**0.0014**
	CC	73 (12.2)	88 (17.5)		1.88 (1.31∼2.73)	0.0006		112 (12)	146 (17.4)		**1.88 (1.4**∼**2.52)**	**0.00002**
	TC+CC	357 (59.5)	346 (68.9)		1.51(1.18∼1.93)	**0.001**		565 (60.5)	584 (69.5)		**1.49 (1.22**∼**1.81)**	**0.00007**

a *P* value was calculated using a χ^2^ test with 2 d.f.

b Adjusted for age, smoking status, education level, age at primiparity, marital status, and HPV infection status. Bold characters indicate the corresponding *P* values, and the ORs were statistically significant after considering FPRP.

Abbreviations: CI, confidence interval; OR, odds ratio.

### 3 Serum IL-16 level and its correlation with the *IL-16* rs1131445 genotype

**Figure 1:**
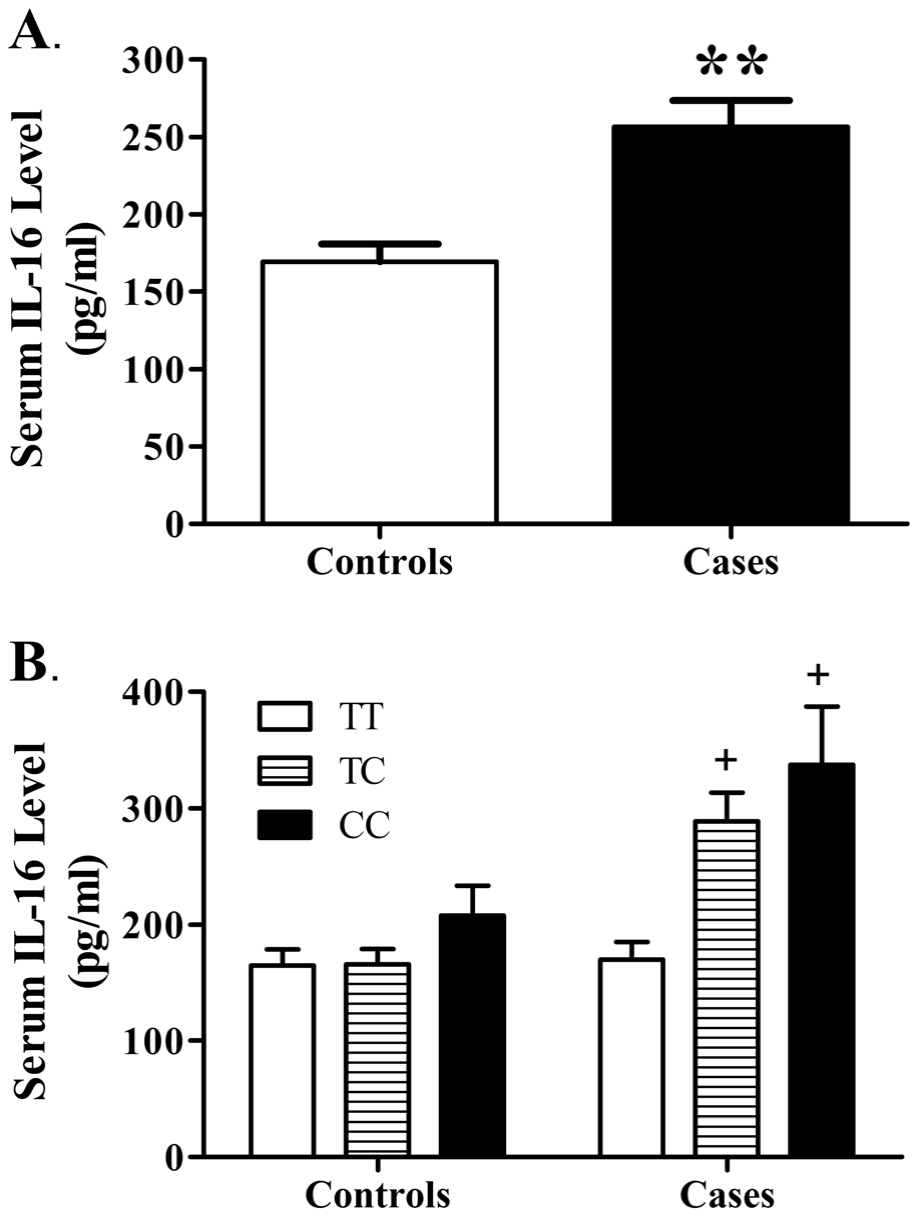
Elevated serum IL-16 levels in patients with cervical cancer and their correlation with the rs1131445 genotype. A. Serum IL-16 levels in controls and patients with cervical cancer, as measured by ELISA. B. Correlations of serum IL-16 levels with genotype in controls and patients. Data were expressed as the mean ± SEM. *P<0.05 vs. controls group. + *P*<0.05 vs. patients with TT genotype.

Given the observed notable association between the rs1131445 and cervical cancer risk, we further investigate the serum IL-16 levels in controls and patients with cervical cancer as well as the potential regulatory effects of the rs1131445 genotype on serum IL-16. We selected 100 patients with cervical cancer and 80 controls from the subjects of Study 2 and examined their serum IL-16 levels by ELISA. The serum IL-16 of patients with cervical cancer significantly increased compared to controls (*P* = 0.001, Figure 1A). Moreover, in patients with cervical cancer, there was a marked correlation between the serum IL-16 and rs1131445 genotype (*P* = 0.001). The rs1131445 C allele-carrying patients had a higher serum IL-16 than the non-carriers (*P*<0.05, Figure 1B). Furthermore, we did not note any statistically significant association of serum IL-16 with the clinical stage and histological type of the cervical cancer (data not shown).

### 4 The effect of rs1131445 on the interaction between miR-135b and *IL-16* 3′-UTR

**Figure 2:**
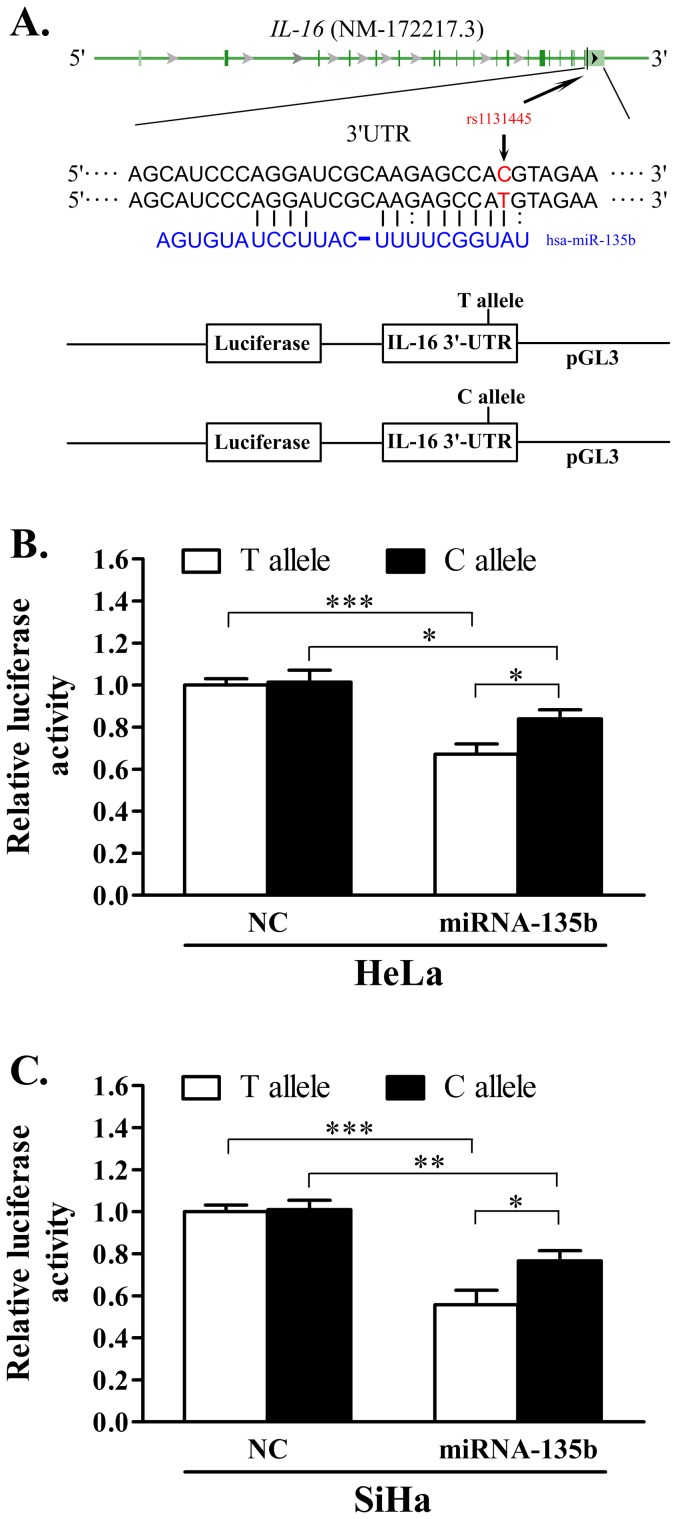
The effect of SNP rs1131445 on the interaction between the *IL-16* 3′-UTR and miR-135b. A. Schema of *IL-16* 3′-UTR harboring a putative miR-135b binding site, the position of rs1131445, and the construct of pGL3-IL-16-3′-UTR-T/C. B and C. Relative luciferase activity in the presence of miR-135b or the negative control miRNA is shown for the *IL-16* 3′-UTR with the T allele and C allele in the HeLa (B) and SiHa (C) cancer cells; data are shown as the percentage relative to the luciferase activity of the cells transfected with the NC and T allele; the error bar represents s.e. from three independent experiments; *P<0.05, **P<0.05, ***P<0.01. NC represents the negative control.

A putative binding site within the 3′-UTR of *IL-16* for miR-135b has been identified using miRBase, miRanda, and PicTar software. It was also predicted that the substitution from the T to the C allele in rs1131445 could decrease the Gibbs binding free energy of miRNA-mRNA by 3.93 KJ/mol [16], which suggests that this SNP could affect the *IL-16* gene expression by altering the miRNA-mRNA binding affinity (Figure 2A). To test this hypothesis, a luciferase assay was performed in the HeLa and SiHa cervical cancer cell lines, which transfected with the pGL3-C-allele or pGL3-T-allele constructs accompanied by chemically synthesized miRNAs miR-135b or a negative control miRNA (Figure 2A). For both constructs, in the presence of miR-135b mimics, the expression of luciferase was significantly reduced (Figure 2B-C), which confirms the functional potential of the miRNA-mRNA interaction. In contrast, the negative control miRNA with no predicting binding site in the *IL-16* 3′-UTR has no effect on the luciferase expression (Figure 2B–C). Moreover, we found that the C allele resulted in a modest but statistically significant increase of luciferase expression compared to that of the T allele (Figure 2B–C).

## Discussion

In the present study, we screened out a cervical cancer susceptibility variant from the miRNA-binding site SNPs within 37 cancer-related genes by a two-stage study. We found that the SNP rs1131445 C allele in the 3′-UTR of *IL-16* was associated with an increased risk of cervical cancer. Using a luciferase assay, we found that miRNA-135b markedly decreased the luciferase expression of pGL3-IL-16-3′-UTR constructs in the Hela and SiHa cervical cancer cell lines. More importantly, these inhibitory effects of miRNA-135b were weakened by the substitution from the T to the C allele in rs1131445. These results support the hypothesis that the variations in the putative miRNA target sites could constitute a susceptibility factor for cervical cancers.

IL-16 is synthesized by various immune and parenchymal cells, including CD4 and CD8 T cells, eosinophils, macrophage/dendritic cells, mast cells, bronchial epithelium, and fibroblasts [27]. The protein directly translated from *IL-16* mRNA is a precursor molecule and must be cleaved into two fragments by caspase-3. The cleaved bioactive IL-16 is released following stimulation by histamine or serotonin and acts as a chemoattractant factor to regulate the inflammatory response [27]. Recently, IL-16 has been recognized as an important promoting factor for tumors [28], [29], [30], [31]. The production of IL-16 correlates with the onset and progression of various hematopoietic cancers and solid cancers [28]. The constitutive IL-16 expression has also been found to upregulate in gliomas [29] and prostate cancer [31]. Moreover, its expression in prostate cancer tissue was correlated to tumor aggressiveness and biochemical relapse of the disease [31]. Serum IL-16 level elevation has been observed in multiple cancers [32], [33]. Serum IL-16 is a predictor for the development of distant metastases of breast cancer [32]. Moreover, in breast cancer, the upregulation of secreted IL-16 enhances the infiltration of monocytes into the tumor tissue [30].

The roles of IL-16 in cervical cancer pathophysiology remain largely unclear. Our findings of serum IL-16 elevation following cervical carcinogenesis and the notable association of *IL-16* with cervical cancer risk suggest that IL-16 could regulate cervical carcinogenesis. This hypothesis is supported by the carcinogenic effects of persistent inflammation on cervix uteri. Immune cell infiltration into tumors is a critical determinant for cervical carcinogenesis [30], [34]. The macrophage infiltrating in cervix uteri increases linearly with cervical disease progression, from normal cervix, low-grade to high grade squamous intraepithelial lesions, to invasive cancer [35]. Tumor-associated macrophages contribute to tumor progression by exerting their multiple functions in tumor cell proliferation, tumor cell invasion, and tumor angiogenesis [36]. Besides macrophages, CD4^+^ regulatory T cells can contribute to carcinogenesis by curtailing the efficacy of T cell immune responses against cancers [37]. In cervical cancer, HPV-specific CD4^+^ T cells suppress cytokine (IFN-γ, IL-2) production to interfere with the anti-tumor immune response [38]. IL-16 can be expressed by epithelial cells under inflammatory conditions [39] and serves to chemoattract monocyptes/macrophages and CD4^+^ T cells to the infection site [40]. Moreover, secreted IL-16 stimulates monocyptes/macrophages to produce various pro-inflammatory cytokines [41]. Therefore, it is plausible that the upregulation of IL-16 in HPV-induced inflammation could promote carcinogenesis by the hyperfunction of macrophages, HPV-specific CD4^+^ T cells, or other inflammatory cells.

Here, we provide the first evidence that rs1131445 in the miR-135b binding site of *IL-16* 3′-UTR, which can affect IL-16 protein expression by interfering with miR135b suppressive function, was significantly associated with the risk of cervical cancer. A functional assay in vitro indicated that the transfection of HeLa and SiHa cells carrying the *IL-16* 3′-UTR with miR-135b mimic significantly reduces the expression of luciferase, which suggests that IL-16 might be targeted by miR-135b. The rs1131445 T to C change inhibits the interaction of miR-135b with *IL-16* 3′-UTR and upregulates its expression. Consistent with this speculation, in vivo analysis revealed that patients who carry the rs1131445 C allele had a higher serum IL-16 than non-carriers, which led to an elevated serum IL-16 of patients compared to healthy controls. The upregulation of miR-135b has been found in cervical cancer [42], which could inhibit the expression of *IL-16* by targeting its 3′-UTR. The rs1131445 C allele impairs the binding of miR-135b to the target and leads to higher constitutive expression of *IL-16* and subsequent release to serum. Given the important regulatory roles of IL-16 release in the immune response and the consequent level of the local inflammatory microenvironment, individuals who carry the rs3134615 C allele would be expected to have elevated risk for cervical cancer. In conclusion, our data suggested that rs1131445, as a functional variant, could modulate the individual risk of cervical cancer likely by deregulating the post-transcriptional regulation of miRNA-135b on *IL-16* expression. Although our study used reasonable numbers of patients and controls and rigorous statistical methods, we could not completely rule out the possibility that spurious results emerged from our case-control association study design due to chance or population stratification. Therefore, further large scale replication study using different ethnic populations, or family-based analysis, or prospective cohort design is required to produce very conclusive results. Additional functional analyses are also required to clarify the exact roles of IL-16 in cervical cancer genesis and progression in the future.

## References

[1] Jemal A, Bray F (2011) Center MM, Ferlay J, Ward E, et al (2011) Global cancer statistics. CA Cancer J Clin 61: 69–90. 21296855 10.3322/caac.20107

[2] Walboomers JM, Jacobs MV, Manos MM, Bosch FX, Kummer JA, et al (1999) Human papillomavirus is a necessary cause of invasive cervical cancer worldwide. J Pathol 189: 12–19. 10451482 10.1002/(SICI)1096-9896(199909)189:1<12::AID-PATH431>3.0.CO;2-F

[3] McCredie MR, Sharples KJ, Paul C, Baranyai J, Medley G, et al (2008) Natural history of cervical neoplasia and risk of invasive cancer in women with cervical intraepithelial neoplasia 3: a retrospective cohort study. Lancet Oncol 9: 425–434. 18407790 10.1016/S1470-2045(08)70103-7

[4] Wang SS, Hildesheim A (2003) Chapter 5: Viral and host factors in human papillomavirus persistence and progression. J Natl Cancer Inst Monogr: 35–40. 10.1093/oxfordjournals.jncimonographs.a003480 12807943

[5] Wang SS, Zuna RE, Wentzensen N, Dunn ST, Sherman ME, et al (2009) Human papillomavirus cofactors by disease progression and human papillomavirus types in the study to understand cervical cancer early endpoints and determinants. Cancer Epidemiol Biomarkers Prev 18: 113–120. 19124488 10.1158/1055-9965.EPI-08-0591 PMC2952430

[6] Magnusson PK, Sparen P, Gyllensten UB (1999) Genetic link to cervical tumours. Nature 400: 29–30. 10403244 10.1038/21801

[7] Safaeian M, Hildesheim A, Gonzalez P, Yu K, Porras C, et al (2012) Single nucleotide polymorphisms in the PRDX3 and RPS19 and risk of HPV persistence and cervical precancer/cancer. PLoS One 7: e33619. 22496757 10.1371/journal.pone.0033619 PMC3322120

[8] Ferguson R, Ramanakumar AV, Koushik A, Coutlee F, Franco E, et al (2012) Human leukocyte antigen G polymorphism is associated with an increased risk of invasive cancer of the uterine cervix. Int J Cancer 131: E312–319. 22095460 10.1002/ijc.27356

[9] Ryan BM, Robles AI, Harris CC (2010) Genetic variation in microRNA networks: the implications for cancer research. Nat Rev Cancer 10: 389–402. 20495573 10.1038/nrc2867 PMC2950312

[10] Carthew RW (2006) Gene regulation by microRNAs. Curr Opin Genet Dev 16: 203–208. 16503132 10.1016/j.gde.2006.02.012

[11] Compagni A, Christofori G (2000) Recent advances in research on multistage tumorigenesis. Br J Cancer 83: 1–5. 10.1054/bjoc.2000.1309 PMC2374546 10883659

[12] Deftereos G, Corrie SR, Feng Q, Morihara J, Stern J, et al (2011) Expression of mir-21 and mir-143 in cervical specimens ranging from histologically normal through to invasive cervical cancer. PLoS One 6: e28423. 22194833 10.1371/journal.pone.0028423 PMC3237431

[13] Xie H, Zhao Y, Caramuta S, Larsson C, Lui WO (2012) miR-205 expression promotes cell proliferation and migration of human cervical cancer cells. PLoS One 7: e46990. 23056551 10.1371/journal.pone.0046990 PMC3463520

[14] Peng RQ, Wan HY, Li HF, Liu M, Li X, et al (2012) MicroRNA-214 suppresses growth and invasiveness of cervical cancer cells by targeting UDP-N-acetyl-alpha-D-galactosamine:polypeptide N-acetylgalactosaminyltransferase 7. J Biol Chem 287: 14301–14309. 22399294 10.1074/jbc.M111.337642 PMC3340176

[15] Ziebarth JD, Bhattacharya A, Chen A, Cui Y (2012) PolymiRTS Database 2.0: linking polymorphisms in microRNA target sites with human diseases and complex traits. Nucleic Acids Res 40: D216–221. 22080514 10.1093/nar/gkr1026 PMC3245163

[16] Landi D, Gemignani F, Naccarati A, Pardini B, Vodicka P, et al (2008) Polymorphisms within micro-RNA-binding sites and risk of sporadic colorectal cancer. Carcinogenesis 29: 579–584. 18192692 10.1093/carcin/bgm304

[17] Naccarati A, Pardini B, Stefano L, Landi D, Slyskova J, et al (2012) Polymorphisms in miRNA-binding sites of nucleotide excision repair genes and colorectal cancer risk. Carcinogenesis 33: 1346–1351. 22581836 10.1093/carcin/bgs172

[18] Brendle A, Lei H, Brandt A, Johansson R, Enquist K, et al (2008) Polymorphisms in predicted microRNA-binding sites in integrin genes and breast cancer: ITGB4 as prognostic marker. Carcinogenesis 29: 1394–1399. 18550570 10.1093/carcin/bgn126

[19] Guan X, Liu Z, Liu H, Yu H, Wang LE, et al (2013) A functional variant at the miR-885-5p binding site of CASP3 confers risk of both index and second primary malignancies in patients with head and neck cancer. FASEB J 27: 1404–1412. 23271051 10.1096/fj.12-223420 PMC3606531

[20] Shi TY, Cheng X, Yu KD, Sun MH, Shao ZM, et al (2013) Functional variants in TNFAIP8 associated with cervical cancer susceptibility and clinical outcomes. Carcinogenesis 34: 770–778. 23299407 10.1093/carcin/bgt001

[21] Lu Y, Yi Y, Liu P, Wen W, James M, et al (2007) Common human cancer genes discovered by integrated gene-expression analysis. PLoS One 2: e1149. 17989776 10.1371/journal.pone.0001149 PMC2065803

[22] Jurinke C, van den Boom D, Cantor CR, Koster H (2002) Automated genotyping using the DNA MassArray technology. Methods Mol Biol 187: 179–192. 12013745 10.1385/1-59259-273-2:179

[23] Scherer WF, Syverton JT, Gey GO (1953) Studies on the propagation in vitro of poliomyelitis viruses. IV. Viral multiplication in a stable strain of human malignant epithelial cells (strain HeLa) derived from an epidermoid carcinoma of the cervix. J Exp Med 97: 695–710. 13052828 10.1084/jem.97.5.695 PMC2136303

[24] Friedl F, Kimura I, Osato T, Ito Y (1970) Studies on a new human cell line (SiHa) derived from carcinoma of uterus. I. Its establishment and morphology. Proc Soc Exp Biol Med 135: 543–545. 5529598 10.3181/00379727-135-35091a

[25] Dupont WD, Plummer WD Jr (1998) Power and sample size calculations for studies involving linear regression. Control Clin Trials 19: 589–601. 9875838 10.1016/s0197-2456(98)00037-3

[26] Wacholder S, Chanock S, Garcia-Closas M, El Ghormli L, Rothman N (2004) Assessing the probability that a positive report is false: an approach for molecular epidemiology studies. J Natl Cancer Inst 96: 434–442. 15026468 10.1093/jnci/djh075 PMC7713993

[27] Cruikshank W, Little F (2008) lnterleukin-16: the ins and outs of regulating T-cell activation. Crit Rev Immunol 28: 467–483. 19265505 10.1615/critrevimmunol.v28.i6.10

[28] Kovacs E (2001) The serum levels of IL-12 and IL-16 in cancer patients. Relation to the tumour stage and previous therapy. Biomed Pharmacother 55: 111–116. 11293814 10.1016/s0753-3322(00)00023-8

[29] Liebrich M, Guo LH, Schluesener HJ, Schwab JM, Dietz K, et al (2007) Expression of interleukin-16 by tumor-associated macrophages/activated microglia in high-grade astrocytic brain tumors. Arch Immunol Ther Exp (Warsz) 55: 41–47. 17221335 10.1007/s00005-007-0003-0 PMC3234149

[30] Milke L, Schulz K, Weigert A, Sha W, Schmid T, et al (2013) Depletion of tristetraprolin in breast cancer cells increases interleukin-16 expression and promotes tumor infiltration with monocytes/macrophages. Carcinogenesis 34: 850–857. 23241166 10.1093/carcin/bgs387

[31] Comperat E, Roupret M, Drouin SJ, Camparo P, Bitker MO, et al (2010) Tissue expression of IL16 in prostate cancer and its association with recurrence after radical prostatectomy. Prostate 70: 1622–1627. 20687232 10.1002/pros.21197

[32] Carlsson A, Wingren C, Kristensson M, Rose C, Ferno M, et al (2011) Molecular serum portraits in patients with primary breast cancer predict the development of distant metastases. Proc Natl Acad Sci U S A 108: 14252–14257. 21844363 10.1073/pnas.1103125108 PMC3161545

[33] Yellapa A, Bahr JM, Bitterman P, Abramowicz JS, Edassery SL, et al (2012) Association of interleukin 16 with the development of ovarian tumor and tumor-associated neoangiogenesis in laying hen model of spontaneous ovarian cancer. Int J Gynecol Cancer 22: 199–207. 22274315 10.1097/IGC.0b013e318236a27b

[34] Boccardo E, Lepique AP, Villa LL (2010) The role of inflammation in HPV carcinogenesis. Carcinogenesis 31: 1905–1912. 20819779 10.1093/carcin/bgq176

[35] Hammes LS, Tekmal RR, Naud P, Edelweiss MI, Kirma N, et al (2007) Macrophages, inflammation and risk of cervical intraepithelial neoplasia (CIN) progression-clinicopathological correlation. Gynecol Oncol 105: 157–165. 17229459 10.1016/j.ygyno.2006.11.023

[36] Fukuda K, Kobayashi A, Watabe K (2012) The role of tumor-associated macrophage in tumor progression. Front Biosci (Schol Ed) 4: 787–798. 22202090 10.2741/s299

[37] Hiura T, Kagamu H, Miura S, Ishida A, Tanaka H, et al (2005) Both regulatory T cells and antitumor effector T cells are primed in the same draining lymph nodes during tumor progression. J Immunol 175: 5058–5066. 16210609 10.4049/jimmunol.175.8.5058

[38] van der Burg SH, Piersma SJ, de Jong A, van der Hulst JM, Kwappenberg KM, et al (2007) Association of cervical cancer with the presence of CD4+ regulatory T cells specific for human papillomavirus antigens. Proc Natl Acad Sci U S A 104: 12087–12092. 17615234 10.1073/pnas.0704672104 PMC1924590

[39] Bellini A, Yoshimura H, Vittori E, Marini M, Mattoli S (1993) Bronchial epithelial cells of patients with asthma release chemoattractant factors for T lymphocytes. J Allergy Clin Immunol 92: 412–424. 8360392 10.1016/0091-6749(93)90120-5

[40] Cruikshank W (1982) Center DM (1982) Modulation of lymphocyte migration by human lymphokines. II. Purification of a lymphotactic factor (LCF). J Immunol 128: 2569–2574. 7042841

[41] Mathy NL, Scheuer W, Lanzendorfer M, Honold K, Ambrosius D, et al (2000) Interleukin-16 stimulates the expression and production of pro-inflammatory cytokines by human monocytes. Immunology 100: 63–69. 10809960 10.1046/j.1365-2567.2000.00997.x PMC2326980

[42] A Gardiner WCM Jr, R Edwards, MAustin, J Lesnock, R Bhargava, et al (2010) MicroRNA analysis in human papillomavirus (HPV)-associated cervical neoplasia and cancer. Infectious Agents and Cancer 5: A55.

